# The effect of a low-fat, plant-based lifestyle intervention (CHIP) on serum HDL levels and the implications for metabolic syndrome status – a cohort study

**DOI:** 10.1186/1743-7075-10-58

**Published:** 2013-10-01

**Authors:** Lillian Kent, Darren Morton, Paul Rankin, Ewan Ward, Ross Grant, John Gobble, Hans Diehl

**Affiliations:** 1 Avondale College of Higher Education, 582 Freemans Drive (PO BOX 19), Cooranbong NSW 2265, Australia; 2 Australasian Research Institute, 185 Fox Valley Rd, Wahroonga, NSW 2076, Australia; 3 Medical Nutrition Therapy Northwest, 13568 SE 97th Ave. Suite 203 Clackamas, Oregon 97015, USA; 4 Lifestyle Medicine Institute, PO Box 818, Loma Linda, CA 92354, USA

**Keywords:** Metabolic Syndrome, Cardiovascular disease, HDL, Lipids, Lifestyle intervention, CHIP

## Abstract

**Background:**

Low levels of high-density lipoproteins (HDL) are considered an important risk factor for cardiovascular disease and constitute one of the criteria for the Metabolic Syndrome (MetS). Lifestyle interventions promoting a low-fat, plant-based eating pattern appear to paradoxically reduce cardiovascular risk but also HDL levels. This study examined the changes in MetS risk factors, in particular HDL, in a large cohort participating in a 30-day lifestyle intervention that promoted a low-fat, plant-based eating pattern.

**Methods:**

Individuals (n = 5,046; mean age = 57.3 ± 12.9 years; 33.5% men, 66.5% women) participating in a in a Complete Health Improvement Program (CHIP) lifestyle intervention within the United States were assessed at baseline and 30 days for changes in body mass index (BMI), blood pressure (BP), lipid profile and fasting plasma glucose (FPG).

**Results:**

HDL levels decreased by 8.7% (p<0.001) despite significant reductions (p<0.001) in BMI (-3.2%), systolic BP (-5.2%), diastolic BP (-5.2%), triglycerides (TG; -7.7%), FPG (-6.3%), LDL (-13.0%), total cholesterol (TC, -11.1%), TC: HDL ratio (-3.2%), and LDL: HDL ratio (-5.3%). While 323 participants classified as having MetS at program entry no longer had this status after the 30 days, 112 participants acquired the MetS classification as a result of reduction in their HDL levels.

**Conclusions:**

When people move towards a low-fat, plant-based diet, HDL levels decrease while other indicators of cardiovascular risk improve. This observation raises questions regarding the value of using HDL levels as a predictor of cardiovascular risk in populations who do not consume a typical western diet. As HDL is part of the assemblage of risk factors that constitute MetS, classifying individuals with MetS may not be appropriate in clinical practice or research when applying lifestyle interventions that promote a plant-based eating pattern.

## Introduction

Epidemiological studies indicate that low levels of plasma or serum HDL is an important risk factor in the development of cardiovascular disease (CVD) [1]. Consequentially, the National Cholesterol Education Program has advocated increasing HDL levels as an important strategy for the primary prevention of CVD [2]. Lowered HDL has also been included as one of the risk factors used to establish a diagnosis of Metabolic Syndrome (MetS) [3].

Lifestyle interventions that promote a low-fat, plant-based eating pattern have been shown to reduce HDL levels [4-8]. Consequentially, it has been suggested that low-fat, plant-based diets may not be ideal for people suffering CVD [9,10], despite being well established to reduce other CVD risk factors including body mass, BP, TC and LDL [4-8]. Furthermore, long-term intervention studies involving low-fat, plant-based diets have even been shown to regress atherosclerotic plaques [4,7] and reduce cardiac events [4], despite also lowering HDL levels. This apparently conflicting observation has led to debate surrounding the merits of a low-fat, plant-based diet for the management of CVD. In a recent review of HDL [11] argued that lowered HDL level is not associated with increased risk of coronary heart disease in the absence of other lipid or non-lipid risk factors.

The purpose of this study was to further explore the changes in CVD risk factors, especially HDL, in a large cohort of individuals participating in a lifestyle intervention that advocated a low-fat, plant-based eating pattern—the Complete Health Improvement Program (CHIP). The effect of these changes on the MetS rubic and the implications of this for classifying individuals with MetS were specifically examined.

## Methods

The study evaluated the pre- to post-biometric changes of 5,046 individuals (mean age = 57.3±12.9 years; 33.5% men, 66.5% women), who self-selected to participate in a CHIP lifestyle intervention conducted in various locations throughout the United States. Consent for the study was obtained from Avondale College of Higher Education Human Research Ethics Committee (Approval No. 20:10:07).

The CHIP intervention, previously described [5,6,12], encouraged and supported participants to move towards a low-fat, plant-based diet *ad libitum*, with emphasis on the whole-food consumption of grains, legumes, fruits and vegetables. Specifically, the program recommended less than 20% of calories be derived from fat. In addition, participants were encouraged to consume 2-2.5 litres of water daily and limit their daily intake of added sugar, sodium and cholesterol to 40 g, 2,000 mg, and 50 mg respectively. Furthermore, the program encouraged participants to engage in 30 minutes of daily moderate-intensity physical activity and practice stress management techniques.

Before participating in the CHIP intervention (baseline) and then again at 30 days (post-intervention), participants’ height, weight, and BP were taken. In addition, fasting (12-hour) blood samples were collected by trained phlebotomists and analysed for TC, LDL, HDL, TG and FPG levels.

The five risk factors for MetS, as described by the "harmonized definition" [3] are: central obesity (based on population specific waist circumference), raised BP (systolic ≥130 mmHg and/or diastolic ≥ 85 mmHg), elevated FPG (≥5.5 mmol/L), increased TG (≥1.7 mmol/L), and decreased HDL (<1.03 mmol/L in males and <1.3 mmol/L in females). The study participants were classified as meeting these criteria at baseline and post-intervention, however, as waist circumference was not measured in this study, BMI >30 kg/m^2^ was used as surrogate for central obesity, as suggested by the International Diabetes Federation [13]. Participants were deemed as having MetS if they met three or more of the defining criteria [3].

The data were analysed using IBM™ Statistics (version 19) and expressed as mean ± standard deviation. The extent of changes (baseline to post-intervention) in the MetS risk factors were assessed using paired t-tests. McNemar chi square test was used to determine changes from baseline to post-intervention in the number of participants who met the five MetS risk factor criteria, as well as the number who were classified as having the syndrome.

## Results

### Overall changes

After 30 days, significant mean reductions (p<0.001) were recorded in all of the five MetS risk factors, including HDL, which decreased almost 9% (Table 1).

**Table 1:** Mean changes in selected risk factors from baseline to 30 days

**Factor**	**Participants (n)**	**Baseline**		**Post-intervention**		**Mean change**	**95% confidence interval**	**% change**	**t statistic**	**p value**
		**Mean**	**SD**	**Mean**	**SD**					
SBP (mmHg)	4550	133.30	19.08	126.35	16.51	-6.95	-7.39, -6.51	-5.2	31.13	<0.001
DBP (mmHg)	4552	79.83	11.04	75.69	9.89	-4.14	-4.43, -3.85	-5.2	28.22	<0.001
BMI (kg/m^2^)	4514	31.01	7.30	30.03	7.01	-0.98	-1.01, -0.96	-3.2	78.11	<0.001
TC (mg/dl)	4655	193.55	41.75	172.09	37.83	-21.46	-22.23, -20.69	-11.1	54.73	<0.001
HDL (mg/dl)	4654	54.84	25.76	50.07	23.16	-4.77	-5.03, -4.51	-8.7	36.56	<0.001
LDL (mg/dl)	4550	131.10	62.02	114.00	54.87	-17.10	-17.90, -16.30	-13.0	41.98	<0.001
TG (mg/dl)	4650	143.35	90.02	132.30	74.55	-11.05	-12.80, -9.31	-7.7	12.41	<0.001
FPG (mg/dl)	4587	101.29	28.94	94.86	20.99	-6.43	-6.96, -5.90	-6.3	23.99	<0.001
TC:HDL	4651	4.01	1.48	3.88	1.40	-0.13	-0.15, -0.11	-3.2	12.00	<0.001
LDL:HDL	4548	2.56	1.04	2.43	0.98	-0.14	-0.15, -0.12	-5.3	15.48	<0.001

### HDL

The change in HDL, stratified according to baseline HDL level, is presented in Table 2. Participants with the highest initial levels of HDL experienced the greatest decreases in the 30 days. The decrease in HDL was not as great as the decrease in LDL and TC (13% and 11%, respectively), resulting in improvements in the TC:HDL ratio of 3% and the LDL:HDL ratio of 5% (Table 1).

**Table 2:** Changes in HDL levels in 30 days according to initial risk factor classification

**HDL level**	**Baseline (n)**	**Post-intervention (n)**	**McNemar chi-squared test (p)**	**Baseline**		**Post-intervention**		**Mean change**	**95% confidence interval**	**% mean change**	**p value**
				**Mean**	**SD**	**Mean**	**SD**				
<40	1196	1676	525 (<0.001)	33.62	4.64	32.69	6.95	-0.94	-1.28, -0.59,	-0.9%	0.546
40-59	2203	2030		48.39	5.53	44.64	7.83	-3.75	-4.02, -3.48,	-7.9%	<0.001
≥60	1255	942		86.39	29.82	76.18	28.53	-10.22	-10.88, -9.56,	-11.8%	<0.001

### MetS

As shown in Table 3, there was a significant reduction in the number of participants who met the MetS criteria for BMI, BP, TG and FPG, but a significant increase in the number of participants who met the criteria for HDL.

**Table 3:** The number of participants meeting metabolic syndrome (MetS) risk factors criteria at baseline and post-intervention

**Risk factor**	**Baseline**	**Post-program**	Improved MetS status*	**% improvement**
	**(N)**	**(N)**	**(N)**	
BMI	2228	1951	+277	12.4%
BP	2761	1994	+767	27.8%
FPG	1618	1145	+472	29.2%
TG	1606	1426	+180	11.2%
HDL	2030	2640	- 610	-30.0%

*Number of participants who improved their MetS status during the intervention for each of the five criteria.

While 1889 (41.7%) participants entered the program characterised as having MetS, this was reduced to 1566 (34.6%) following the intervention; a reduction of 323 participants. Two hundred and fifty seven participants who were not classified as having MetS at program entry acquired this status at the completion of the intervention, however, 157 of these individuals (61%) only did so because of reduced HDL levels. For these individuals, the TC: HDL and LDL: HDL ratios increased significantly from baseline to post-intervention (3.56±0.77 versus 4.12±0.87, p<0.001; 2.31±0.71 versus 2.44±0.76, p=0.006, respectively) as both TC (12%, p<0.001) and LDL (15%, p<0.001) did not decrease as much as HDL (21%, p<0.001).

## Discussion

The most striking observation of the present study is that when people move towards a low-fat, plant-based diet, HDL levels tend to decrease while all other measures of cardiovascular risk improve. This large study is the first to highlight the implications of this effect for MetS classification. Furthermore, the findings raise questions about the usefulness of measuring HDL levels for predicting cardiovascular risk, especially among specific groups such as vegetarian populations.

Further evidence that the cardiovascular risk of the participants in this study improved despite the trend for HDL to decrease, is seen in the improvements in the overall TC: HDL and LDL: HDL ratios. A meta-analysis of 61 prospective studies with vascular deaths as an endpoint suggested that the TC: HDL ratio was more predictive than either HDL or non-HDL cholesterol sub-fractions, and two times more predictive than TC [14]. However, in the present study, this ratio, as well as the ratio of LDL: HDL, significantly increased among participants who were newly characterised with MetS and low HDL levels. In addition, there were significant reductions in the number of participants who met the MetS criteria for BMI, BP, TG and FPG levels (11-29%), as compared to the 30% increase in the number of participants who meet the HDL criterion. These results show that in the context of interventions that emphasise a low-fat, plant-based eating pattern, changes in HDL levels may be misleading, whether measured as an absolute value or calculated as a ratio of TC or LDL.

The consistently strong inverse association between low HDL levels and the risk of cardiovascular events observed in epidemiological studies [15,16], has traditionally been explained by its role in reverse cholesterol transport (RCT), also known as cholesterol efflux [16]. Notwithstanding this role, HDL more recently has been shown to have many anti-atherogenic properties, which include anti-inflammatory, anti-apoptotic, nitric oxide promoting, prostacyclin-stabilizing, and platelet-inhibiting functions [17,18]. Indeed, it has been postulated that the anti-inflammatory properties of HDL and its ability to protect LDL from oxidation may be just as important as its role in RCT [19].

Despite the documented anti-atherogenic properties of HDL, there is evidence that questions the relationship between HDL levels and risk of cardiovascular events, as many individuals who suffer coronary atherosclerotic events have normal or even elevated HDL levels [18,20]. Specifically, the Framingham study demonstrated that more than 40% of cardiac events occurred in men and women with normal HDL levels [20]. In addition, other studies have shown that when HDL levels are raised pharmacologically, the elevated levels do not always correlate with reduced risk of coronary heart disease [21,22].

The result of this study, which suggests that HDL levels may not be helpful for predicting cardiovascular risk in individuals consuming a low-fat, plant-based diet, is supported by other epidemiological and clinical studies. Over 30 years ago, Connor observed that the Tarahumara Indians of Mexico, who consumed a largely plant-based diet comprising approximately 12% fat, 13% protein (predominantly from corn and beans) and 85% carbohydrate, had very low rates of vascular disease and blood lipids, including HDL [23]. However, blood lipids, including HDL, were observed to significantly increase after only five weeks when their traditional diet was changed to a Western diet [24]. It was argued that the increase in HDL was the "normal response to a high-fat diet" and that low-HDL in concert with low-LDL in a low-fat diet is associated with a low risk of coronary disease. Other epidemiological findings also show that individuals who consume a plant-based diet are at lower risk of CVD and type 2 diabetes mellitus, despite having lowered HDL levels [19,25]. In addition, the Lifestyle Heart Trial [7], which incorporated a plant-based diet with less than 10% fat, showed a 7.9% improvement in measured coronary artery percent diameter stenosis after five years despite a 13% reduction in HDL. Similarly, individuals with diagnosed CVD and a recommendation for bypass surgery who participated in the Pritikin residential program, which recommends a plant-based diet (10% fat), experienced a 16% reduction in HDL but decreases in symptomatic angina [26]. These patients averted surgery for more than five years after program entry despite sustained lowered HDL levels.

The value of increasing HDL levels has been further questioned as its range of functions has become better understood [11,17,18]. With its heterogeneous structure and subtypes, varying functions are now being demonstrated [18]. To date, a number of apolipoproteins and antioxidant enzymes have been identified in the HDL structure that may explain its anti-inflammatory (e.g. ApoA-1 and paraoxonase 1) and inflammatory properties (e.g. ApoA-II and ApoC-III) [16,18,27]. Although the mechanisms governing HDL inflammatory and anti-inflammatory ratios are yet to be fully elucidated, these may at least in part be influenced by the presence of oxidized lipids and oxidants, which inhibit or directly damage the anti-inflammatory molecules on HDL [27]. Noteworthy, antioxidants and phytonutrients abundant in plant foods may increase the activity of HDL enzymes or counter the adverse effect of oxidants on apoA-1 and/or the pro-inflammatory effect on LDL lipids [28]. Given that people following a low-fat, plant-based diet typically have lowered levels of serum TC and LDL, the need for elevated HDL levels may be diminished from an RCT perspective. Indeed, lifestyle factors have been shown to influence the subpopulations of HDL.

Additional evidence against the role of HDL in CVD risk has come from genetic studies, designed to examine the associations of LDL, HDL and TG with CVD risk [29,30]. Early studies showed that rare mutations that encode LCAT (lecithin cholesterol acyl transferase) and ABCA1 (ATP-binding cassette transporter, also known as cholesterol efflux regulatory protein (CERP)), which profoundly reduce HDL levels, were inconsistently associated with CVD risk [29]. Subsequent Mendelian randomization (MR) analyses examined several DNA variants that affect HDL levels and found no associations with major adverse CVD end points and risk of myocardial infarction [29]. In contrast, genetics variants that raise LDL levels significantly increased CVD risk. More recent MR analyses, using carotid intima-media thickness (CIMT) as a surrogate for atherosclerosis, confirmed a strong relationship between LDL and CIMT, but not with HDL and TG [30].

There is also growing evidence that lifestyle interventions may be able to modulate the inflammatory or anti-inflammatory properties of HDL. In patients at risk of CVD, the anti-inflammatory properties of HDL improved following lifestyle modification, despite reductions in HDL [28]. In another study, the HDL shifted from pro-inflammatory to anti-inflammatory in obese men, with MetS, who underwent a three-week intervention involving a low-fat, high- fibre diet and exercise [19]. More specifically, consumption of saturated fat reduces the anti-inflammatory potential of HDL, but consumption of polyunsaturated fat has been shown to increase it [31]. The regulation and function of HDL appears more complex than originally thought-- although high HDL levels are associated with reduced CVD at a population level, at an individual level HDL *function* may be more important than the actual HDL *levels*[32].

## Conclusion

The findings of this study question the value of using HDL levels as a predictor of cardiovascular risk, especially in populations who do not consume a typical Western diet. The appropriateness of applying standard HDL criteria to populations that consume a plant-based diet may be particularly problematic. Finally, characterising individuals with MetS may not be appropriate in clinical practice or research when applying lifestyle interventions that promote a plant-based eating pattern.

## Abbreviations

MetS: Metabolic Syndrome; CHIP: Complete Health Improvement Program; HDL: High-density lipoprotein; LDL: Low-density lipoprotein; TC: Total cholesterol; TG: Triglycerides; FPG: Fasting plasma glucose; BMI: Body mass index; SBP: Systolic blood pressure; DBP: Diastolic blood pressure; CVD: Cardiovascular disease; Apo: Apolipoprotein; LCAT: LECITHIN cholesterol acyl transferase; ABCA1: ATP-binding cassette transporter.

## Competing interests

The authors declare that they have no competing interests.

## Authors’ contributions

HD developed the CHIP intervention. DM, PR and LK were involved in the design of this study. JG collected all the data. LK conducted the data analyses. All authors were involved in the interpretation of the analyses, drafting of the manuscript, and critical revision of intellectual content. All authors approved the final version to be published.
